# 1-(2,4-Dichloro­phen­yl)-3-(4-methyl­phen­yl)prop-2-en-1-one

**DOI:** 10.1107/S1600536808011008

**Published:** 2008-04-30

**Authors:** Hoong-Kun Fun, Samuel Robinson Jebas, P. S. Patil, S. M. Dharmaprakash

**Affiliations:** a X-ray Crystallography Unit, School of Physics, Universiti Sains Malaysia, 11800 USM, Penang, Malaysia; b Department of Studies in Physics, Mangalore University, Mangalagangotri, Mangalore 574 199, India

## Abstract

The mol­ecule of the title compound, C_16_H_12_Cl_2_O, adopts an *E* configuration. The dihedral angle between the two benzene rings is 42.09 (5)°. In the crystal structure, mol­ecules are linked into a three-dimensional framework by weak C—H⋯O inter­actions and by C—H⋯π inter­actions involving the methyl­phenyl ring.

## Related literature

For related literature, see: Agrinskaya *et al.* (1999 ▶); Gu *et al.* (2008 ▶); Patil *et al.* (2006 ▶); Patil, Dharmaprakash *et al.* (2007 ▶); Patil, Teh *et al.* (2007 ▶).
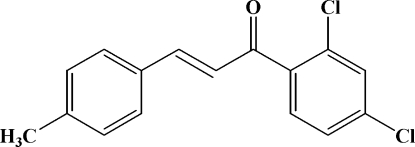


## Experimental

### 

#### Crystal data

C_16_H_12_Cl_2_O
*M* *_r_* = 291.16
Orthorhombic, 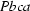

*a* = 12.54850 (1) Å
*b* = 7.47750 (1) Å
*c* = 28.7764 (3) Å
*V* = 2700.13 (3) Å^3^
*Z* = 8
Mo *K*α radiation
μ = 0.47 mm^−1^
*T* = 100.0 (1) K
0.47 × 0.39 × 0.20 mm

#### Data collection

Bruker SMART APEXII CCD area-detector diffractometer
Absorption correction: multi-scan (*SADABS*; Bruker, 2005 ▶) *T*_min_ = 0.811, *T*_max_ = 0.914
50054 measured reflections
7296 independent reflections
4995 reflections with *I* > 2σ(*I*)
*R*_int_ = 0.052

#### Refinement

*R*[*F* ^2^ > 2σ(*F* ^2^)] = 0.045
*wR*(*F* ^2^) = 0.148
*S* = 1.07
7296 reflections
173 parameters
H-atom parameters constrained
Δρ_max_ = 0.64 e Å^−3^
Δρ_min_ = −0.53 e Å^−3^

### 

Data collection: *APEX2* (Bruker, 2005 ▶); cell refinement: *APEX2*; data reduction: *SAINT* (Bruker, 2005 ▶); program(s) used to solve structure: *SHELXTL* (Sheldrick, 2008 ▶); program(s) used to refine structure: *SHELXTL*; molecular graphics: *SHELXTL*; software used to prepare material for publication: *SHELXTL* and *PLATON* (Spek, 2003 ▶).

## Supplementary Material

Crystal structure: contains datablocks global, I. DOI: 10.1107/S1600536808011008/ci2584sup1.cif

Structure factors: contains datablocks I. DOI: 10.1107/S1600536808011008/ci2584Isup2.hkl

Additional supplementary materials:  crystallographic information; 3D view; checkCIF report

## Figures and Tables

**Table 1:** Hydrogen-bond geometry (Å, °)

*D*—H⋯*A*	*D*—H	H⋯*A*	*D*⋯*A*	*D*—H⋯*A*
C2—H2⋯O1^i^	0.93	2.55	3.4352 (15)	159
C8—H8⋯O1^ii^	0.93	2.55	3.1995 (14)	127
C11—H11⋯*Cg*1^iii^	0.93	2.81	3.5611 (13)	139
C14—H14⋯*Cg*1^iv^	0.93	2.93	3.7066 (13)	142

Symmetry codes: (i) 
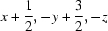
; (ii) 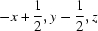; (iii) 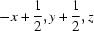; (iv) 
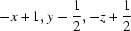
. *Cg*1 is the centroid of the C10–C15 benzene ring.

**Fig. 1.**
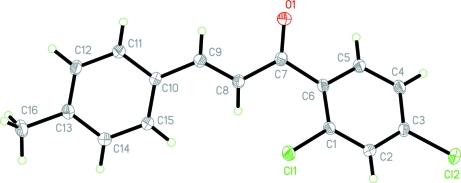
The molecular structure of the title compound, showing 50% probability displacement ellipsoids and the atom numbering scheme.

**Fig. 2.**
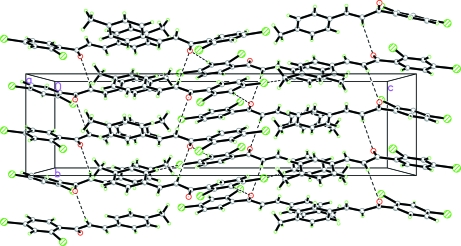
The crystal packing of the title compound, viewed approximately along the a axis. Hydrogen bonds are shown as dashed lines.

## supplementary crystallographic information

### Comment

Chalcone derivatives have been studied extensively owing to their fascinating, technologically relevant nonlinear optical properties (Gu *et al.*, 2008; Agrinskaya *et al.*, 1999; Patil *et al.*, 2006; Patil, Dharmaprakash *et al.*, 2007; Patil, Teh *et al.*, 2007).

The title molecule exhibits an E configuration with respect to the C8═C9 double bond [1.3424 (14) Å]; the C7—C8—-C9—-C10 torsion angle is 179.61 (11)°. The dihedral angle between the two benzene rings is 42.09 (5)°. The bond lengths and angles in the title molecule have normal values.

The crystal structure is stabilized by weak C—H···O intermolecular hydrogen bonding interactions (Table 1), which link the molecules into a three-dimensional framework (Fig. 2). In addition weak C—H..π interactions involving the C10—C15 benzene ring (centroid Cg1) is observed.

### Experimental

The title compound was synthesized by the condensation of p-tolualdehyde (0.01 mol) with 2,4-dichloroacetophenone (0.01 mol) in methanol (60 ml) in the presence of a catalytic amount of sodium hydroxide solution (5 ml, 30%). After stirring for 2 h, the contents of the flask were poured into ice-cold water (500 ml) and left to stand for 5 h. The resulting crude solid was collected by filtration and dried. Crystals suitable for single-crystal X-ray diffraction were grown by slow evaporation of an acetone solution at room temperature.

### Refinement

H atoms were positioned geometrically [C-H = 0.93 (aromatic) or 0.96 Å (methyl)] and refined using a riding model, with *U*_iso_(H) = 1.5*U*_eq_(methyl C) or 1.2U_eq_(C).

### Crystal data

**Table:**

C_16_H_12_Cl_2_O	*F*_000_ = 1200
*M**_r_* = 291.16	*D*_x_ = 1.432 Mg m^−^^3^
Orthorhombic, *P**b**c**a*	Mo *K*α radiation λ = 0.71073 Å
Hall symbol: -P 2ac 2ab	Cell parameters from 9639 reflections
*a* = 12.54850 (1) Å	θ = 2.8–34.6º
*b* = 7.47750 (1) Å	µ = 0.47 mm^−^^1^
*c* = 28.7764 (3) Å	*T* = 100.0 (1) K
*V* = 2700.13 (3) Å^3^	Block, colourless
*Z* = 8	0.47 × 0.39 × 0.20 mm

### Data collection

**Table:**

Bruker SMART APEXII CCD area-detector diffractometer	7296 independent reflections
Radiation source: fine-focus sealed tube	4995 reflections with *I* > 2σ(*I*)
Monochromator: graphite	*R*_int_ = 0.052
*T* = 100.0(1) K	θ_max_ = 37.9º
φ and ω scans	θ_min_ = 1.4º
Absorption correction: multi-scan(SADABS; Bruker, 2005)	*h* = −21→21
*T*_min_ = 0.811, *T*_max_ = 0.914	*k* = −12→12
50054 measured reflections	*l* = −48→49

### Refinement

**Table:**

Refinement on *F*^2^	Secondary atom site location: difference Fourier map
Least-squares matrix: full	Hydrogen site location: inferred from neighbouring sites
*R*[*F*^2^ > 2σ(*F*^2^)] = 0.045	H-atom parameters constrained
*wR*(*F*^2^) = 0.148	*w* = 1/[σ^2^(*F*_o_^2^) + (0.078*P*)^2^ + 0.0443*P*] where *P* = (*F*_o_^2^ + 2*F*_c_^2^)/3
*S* = 1.07	(Δ/σ)_max_ = 0.001
7296 reflections	Δρ_max_ = 0.64 e Å^−^^3^
173 parameters	Δρ_min_ = −0.53 e Å^−^^3^
Primary atom site location: structure-invariant direct methods	Extinction correction: none

### Special details

**Table:**

Experimental. The data was collected with the Oxford Cyrosystem Cobra low-temperature attachment.
Geometry. All e.s.d.'s (except the e.s.d. in the dihedral angle between two l.s. planes) are estimated using the full covariance matrix. The cell e.s.d.'s are taken into account individually in the estimation of e.s.d.'s in distances, angles and torsion angles; correlations between e.s.d.'s in cell parameters are only used when they are defined by crystal symmetry. An approximate (isotropic) treatment of cell e.s.d.'s is used for estimating e.s.d.'s involving l.s. planes.
Refinement. Refinement of *F*^2^ against ALL reflections. The weighted *R*-factor *wR* and goodness of fit *S* are based on *F*^2^, conventional *R*-factors *R* are based on *F*, with *F* set to zero for negative *F*^2^. The threshold expression of *F*^2^ > σ(*F*^2^) is used only for calculating *R*-factors(gt) *etc*. and is not relevant to the choice of reflections for refinement. *R*-factors based on *F*^2^ are statistically about twice as large as those based on *F*, and *R*- factors based on ALL data will be even larger.

### Fractional atomic coordinates and isotropic or equivalent isotropic displacement parameters (Å^2^)

**Table:**

	*x*	*y*	*z*	*U*_iso_*/*U*_eq_	
Cl1	0.46470 (2)	0.76088 (4)	0.057148 (10)	0.02125 (8)	
Cl2	0.36980 (3)	0.56187 (5)	−0.116828 (9)	0.02538 (9)	
O1	0.13288 (7)	0.72551 (12)	0.08499 (3)	0.02032 (17)	
C1	0.37202 (9)	0.67308 (15)	0.01850 (4)	0.0158 (2)	
C2	0.40554 (10)	0.64861 (15)	−0.02709 (4)	0.0177 (2)	
H2	0.4759	0.6696	−0.0356	0.021*	
C3	0.33080 (10)	0.59184 (15)	−0.05947 (4)	0.0177 (2)	
C4	0.22518 (9)	0.56292 (16)	−0.04764 (4)	0.0183 (2)	
H4	0.1760	0.5265	−0.0699	0.022*	
C5	0.19470 (9)	0.58948 (15)	−0.00210 (4)	0.0173 (2)	
H5	0.1238	0.5715	0.0060	0.021*	
C6	0.26690 (9)	0.64245 (14)	0.03220 (3)	0.01531 (19)	
C7	0.22310 (9)	0.66606 (15)	0.08060 (4)	0.0165 (2)	
C8	0.28861 (9)	0.60913 (15)	0.12039 (4)	0.0168 (2)	
H8	0.3512	0.5458	0.1151	0.020*	
C9	0.25956 (10)	0.64712 (15)	0.16419 (4)	0.0168 (2)	
H9	0.1962	0.7100	0.1680	0.020*	
C10	0.31720 (9)	0.59981 (15)	0.20655 (4)	0.01556 (19)	
C11	0.27256 (10)	0.64353 (15)	0.24970 (4)	0.0179 (2)	
H11	0.2075	0.7031	0.2508	0.021*	
C12	0.32405 (10)	0.59919 (16)	0.29076 (4)	0.0189 (2)	
H12	0.2929	0.6294	0.3190	0.023*	
C13	0.42150 (10)	0.51030 (16)	0.29039 (4)	0.0182 (2)	
C14	0.46702 (9)	0.46835 (16)	0.24731 (4)	0.0185 (2)	
H14	0.5325	0.4102	0.2463	0.022*	
C15	0.41598 (9)	0.51215 (16)	0.20617 (4)	0.0177 (2)	
H15	0.4476	0.4831	0.1779	0.021*	
C16	0.47564 (11)	0.45835 (18)	0.33516 (4)	0.0251 (3)	
H16A	0.4802	0.5608	0.3552	0.038*	
H16B	0.5460	0.4149	0.3286	0.038*	
H16C	0.4352	0.3662	0.3503	0.038*	

### Atomic displacement parameters (Å^2^)

**Table:**

	*U*^11^	*U*^22^	*U*^33^	*U*^12^	*U*^13^	*U*^23^
Cl1	0.01778 (15)	0.02973 (16)	0.01623 (13)	−0.00376 (10)	−0.00292 (9)	−0.00163 (10)
Cl2	0.02586 (17)	0.03750 (18)	0.01277 (12)	0.00235 (12)	0.00091 (10)	−0.00154 (10)
O1	0.0168 (4)	0.0256 (4)	0.0185 (4)	0.0022 (3)	−0.0001 (3)	−0.0017 (3)
C1	0.0158 (5)	0.0171 (5)	0.0145 (4)	0.0009 (4)	−0.0025 (4)	−0.0003 (3)
C2	0.0158 (5)	0.0216 (5)	0.0157 (4)	0.0016 (4)	0.0004 (4)	0.0001 (4)
C3	0.0213 (6)	0.0200 (5)	0.0117 (4)	0.0020 (4)	−0.0010 (4)	0.0007 (3)
C4	0.0183 (5)	0.0210 (5)	0.0156 (4)	−0.0001 (4)	−0.0043 (4)	−0.0007 (4)
C5	0.0166 (5)	0.0198 (5)	0.0154 (4)	−0.0007 (4)	−0.0014 (4)	0.0005 (4)
C6	0.0177 (5)	0.0155 (4)	0.0127 (4)	0.0013 (4)	−0.0010 (4)	0.0012 (3)
C7	0.0191 (5)	0.0152 (4)	0.0153 (4)	−0.0006 (4)	0.0005 (4)	−0.0008 (3)
C8	0.0188 (5)	0.0172 (5)	0.0144 (4)	0.0018 (4)	0.0001 (4)	0.0001 (3)
C9	0.0170 (5)	0.0173 (5)	0.0159 (4)	−0.0006 (4)	0.0003 (4)	−0.0001 (3)
C10	0.0177 (5)	0.0163 (4)	0.0127 (4)	−0.0004 (4)	0.0012 (4)	−0.0001 (3)
C11	0.0180 (5)	0.0200 (5)	0.0156 (4)	0.0021 (4)	0.0006 (4)	−0.0008 (4)
C12	0.0222 (6)	0.0218 (5)	0.0126 (4)	0.0004 (4)	0.0013 (4)	−0.0008 (4)
C13	0.0204 (6)	0.0184 (5)	0.0158 (5)	−0.0024 (4)	−0.0019 (4)	0.0007 (4)
C14	0.0168 (5)	0.0207 (5)	0.0179 (5)	0.0013 (4)	0.0002 (4)	0.0001 (4)
C15	0.0175 (5)	0.0209 (5)	0.0149 (4)	0.0013 (4)	0.0009 (4)	−0.0002 (4)
C16	0.0284 (7)	0.0290 (6)	0.0179 (5)	0.0016 (5)	−0.0058 (5)	0.0011 (4)

### Geometric parameters (Å, °)

**Table:**

Cl1—C1	1.7380 (11)	C9—C10	1.4609 (15)
Cl2—C3	1.7360 (11)	C9—H9	0.93
O1—C7	1.2228 (14)	C10—C11	1.4008 (15)
C1—C2	1.3895 (15)	C10—C15	1.4022 (17)
C1—C6	1.3957 (16)	C11—C12	1.3871 (16)
C2—C3	1.3887 (16)	C11—H11	0.93
C2—H2	0.93	C12—C13	1.3919 (18)
C3—C4	1.3854 (17)	C12—H12	0.93
C4—C5	1.3795 (15)	C13—C14	1.4006 (16)
C4—H4	0.93	C13—C16	1.5072 (16)
C5—C6	1.3971 (15)	C14—C15	1.3855 (16)
C5—H5	0.93	C14—H14	0.93
C6—C7	1.5077 (15)	C15—H15	0.93
C7—C8	1.4725 (15)	C16—H16A	0.96
C8—C9	1.3424 (14)	C16—H16B	0.96
C8—H8	0.93	C16—H16C	0.96
			
C2—C1—C6	122.08 (10)	C10—C9—H9	116.6
C2—C1—Cl1	116.83 (9)	C11—C10—C15	118.00 (10)
C6—C1—Cl1	120.90 (8)	C11—C10—C9	119.02 (10)
C3—C2—C1	118.00 (11)	C15—C10—C9	122.98 (10)
C3—C2—H2	121.0	C12—C11—C10	120.88 (11)
C1—C2—H2	121.0	C12—C11—H11	119.6
C4—C3—C2	121.92 (10)	C10—C11—H11	119.6
C4—C3—Cl2	118.90 (9)	C11—C12—C13	121.12 (10)
C2—C3—Cl2	119.15 (10)	C11—C12—H12	119.4
C5—C4—C3	118.45 (10)	C13—C12—H12	119.4
C5—C4—H4	120.8	C12—C13—C14	118.17 (10)
C3—C4—H4	120.8	C12—C13—C16	120.83 (10)
C4—C5—C6	122.15 (11)	C14—C13—C16	120.99 (11)
C4—C5—H5	118.9	C15—C14—C13	121.00 (11)
C6—C5—H5	118.9	C15—C14—H14	119.5
C1—C6—C5	117.38 (10)	C13—C14—H14	119.5
C1—C6—C7	125.90 (10)	C14—C15—C10	120.83 (10)
C5—C6—C7	116.70 (10)	C14—C15—H15	119.6
O1—C7—C8	122.79 (10)	C10—C15—H15	119.6
O1—C7—C6	118.40 (10)	C13—C16—H16A	109.5
C8—C7—C6	118.75 (10)	C13—C16—H16B	109.5
C9—C8—C7	121.15 (11)	H16A—C16—H16B	109.5
C9—C8—H8	119.4	C13—C16—H16C	109.5
C7—C8—H8	119.4	H16A—C16—H16C	109.5
C8—C9—C10	126.71 (11)	H16B—C16—H16C	109.5
C8—C9—H9	116.6		
			
C6—C1—C2—C3	0.00 (17)	C5—C6—C7—C8	141.55 (11)
Cl1—C1—C2—C3	−175.09 (9)	O1—C7—C8—C9	−11.25 (18)
C1—C2—C3—C4	1.20 (17)	C6—C7—C8—C9	171.61 (10)
C1—C2—C3—Cl2	179.44 (9)	C7—C8—C9—C10	−179.61 (11)
C2—C3—C4—C5	−0.88 (17)	C8—C9—C10—C11	−177.16 (12)
Cl2—C3—C4—C5	−179.12 (9)	C8—C9—C10—C15	2.91 (19)
C3—C4—C5—C6	−0.67 (17)	C15—C10—C11—C12	−0.83 (17)
C2—C1—C6—C5	−1.44 (17)	C9—C10—C11—C12	179.24 (11)
Cl1—C1—C6—C5	173.45 (9)	C10—C11—C12—C13	0.09 (19)
C2—C1—C6—C7	−179.70 (10)	C11—C12—C13—C14	0.69 (18)
Cl1—C1—C6—C7	−4.80 (16)	C11—C12—C13—C16	−178.42 (11)
C4—C5—C6—C1	1.79 (17)	C12—C13—C14—C15	−0.73 (18)
C4—C5—C6—C7	−179.80 (10)	C16—C13—C14—C15	178.38 (11)
C1—C6—C7—O1	142.55 (12)	C13—C14—C15—C10	−0.01 (18)
C5—C6—C7—O1	−35.72 (15)	C11—C10—C15—C14	0.79 (17)
C1—C6—C7—C8	−40.19 (15)	C9—C10—C15—C14	−179.29 (11)

### Hydrogen-bond geometry (Å, °)

**Table:**

*D*—H···*A*	*D*—H	H···*A*	*D*···*A*	*D*—H···*A*
C2—H2···O1^i^	0.93	2.55	3.4352 (15)	159
C8—H8···O1^ii^	0.93	2.55	3.1995 (14)	127
C11—H11···Cg1^iii^	0.93	2.81	3.5611 (13)	139
C14—H14···Cg1^iv^	0.93	2.93	3.7066 (13)	142

Symmetry codes: (i) *x*+1/2, −*y*+3/2, −*z*; (ii) −*x*+1/2, *y*−1/2, *z*; (iii) −*x*+1/2, *y*+1/2, *z*; (iv) −*x*+1, *y*−1/2, −*z*+1/2.

## References

[bb1] Agrinskaya, N. V., Lukoshkin, V. A., Kudryavtsev, V. V., Nosova, G. I., Solovskaya, N. A. & Yakimanski, A. V. (1999). *Phys. Solid State*, **41**, 1914–1917.

[bb2] Bruker (2005). *APEX2*, *SAINT* and *SADABS* Bruker AXS Inc., Madison, Wisconsin, USA.

[bb3] Gu, B., Ji, W., Patil, P. S., Dharmaprakash, S. M. & Wang, H. T. (2008). *Appl. Phys. Lett.* **92**, 091118–091120.

[bb4] Patil, P. S., Dharmaprakash, S. M., Fun, H.-K. & Karthikeyan, M. S. (2006). *J. Cryst. Growth*, **297**, 111–116.

[bb5] Patil, P. S., Dharmaprakash, S. M., Ramakrishna, K., Fun, H.-K., Sai Santosh Kumar, R. & Rao, D. N. (2007). *J. Cryst. Growth*, **303**, 520–524.

[bb6] Patil, P. S., Teh, J. B.-J., Fun, H.-K., Razak, I. A. & Dharmaprakash, S. M. (2007). *Acta Cryst.* E**63**, o2122–o2123.

[bb7] Sheldrick, G. M. (2008). *Acta Cryst.* A**64**, 112–122. 10.1107/S0108767307043930 18156677

[bb8] Spek, A. L. (2003). *J. Appl. Cryst.* **36**, 7–13.

